# Science and policy in extremis: the UK’s initial response to COVID-19

**DOI:** 10.1007/s13194-021-00407-z

**Published:** 2021-08-25

**Authors:** Jonathan Birch

**Affiliations:** Department of Philosophy, Logic and Scientific Method, London School of Economics and Political Science, Houghton Street, London, WC2A 2AE UK

**Keywords:** Science and policy, Science and values, Scientific advice, COVID-19

## Abstract

Drawing on the SAGE minutes and other documents, I consider the wider lessons for norms of scientific advising that can be learned from the UK’s initial response to coronavirus in the period January–March 2020, when an initial strategy that planned to avoid total suppression of transmission was abruptly replaced by an aggressive suppression strategy. I introduce a distinction between “normatively light advice”, in which no specific policy option is recommended, and “normatively heavy advice” that does make an explicit recommendation. I argue that, although scientific advisers should avoid normatively heavy advice in normal times in order to facilitate democratic accountability, this norm can be permissibly overridden in situations of grave emergency. SAGE’s major mistake in early 2020 was not that of endorsing a particular strategy, nor that of being insufficiently precautionary, but that of relying too heavily on a specific set of “reasonable worst-case” planning assumptions. I formulate some proposals that assign a more circumscribed role to “worst-case” thinking in emergency planning. In an epilogue, I consider what the implications of my proposals would have been for the UK’s response to the “second wave” of late 2020.

## Studying the table: science, policy and the role of philosophy of science

The COVID-19 pandemic has presented governments around the world with extraordinarily difficult decisions. Governments have generally sought to base these decisions on scientific advice, but there are many ways in which a decision can be “based on scientific advice”. My focus here is on the United Kingdom, and on the UK's initial response to COVID-19 in the early months of 2020. My aim is to analyse the advisory and decision-making process.^1^

I will not provide a detailed narrative account here,^2^ but I will set the scene briefly. On 13 January 2020, the UK government’s “New and Emerging Respiratory Virus Threats Advisory Group” (NERVTAG) held an “extraordinary meeting” to discuss a new pathogen: the “Wuhan novel coronavirus”, subsequently named SARS-CoV-2. Although the group considered the risk to the UK population to be “very low” (NERVTAG, 2020), the threat was considered serious enough to convene the UK’s most important scientific advisory body, the “Scientific Advisory Group for Emergencies” (SAGE). On 22 January 2020, the first “precautionary” meeting of SAGE on the new coronavirus took place at 10 Victoria Street, London.

The severity of the threat, at this time, was extremely uncertain. The minutes note, optimistically, that “SAGE is unable to say at this stage whether it might be required to reconvene” (SAGE, 2020k). By the end of March 2020, SAGE had met twenty-one times—and the daily lives of everyone in the country had changed radically, partly as a result of its advice. The UK was in the grip of the first wave of a terrible epidemic. Official death counts would soon reach a 7-day average of over 900 per day (UK Government, 2021a). A national lockdown introduced on 23 March had confined all but essential workers to their homes. SAGE itself could no longer meet in person and was meeting via Zoom.

The dramatic policy interventions of late March arrived at a time when infection was already widespread. On 2 March, a subgroup of SAGE (the “Scientific Pandemic Influence subgroup on Modelling”, SPI-M-O) had reported that “It is highly likely that there is sustained transmission of COVID-19 in the UK at present” (SPI-M-O, 2020a). In the absence of mass testing, certainty was impossible—and SAGE had, in February, advised that mass testing would be unfeasible.^3^ But retrospective modelling now estimates that the cumulative number of COVID-19 infections in England was in the range 20–28,000 on 2 March (Birrell et al., 2021). Levels of infection continued to grow exponentially throughout the first half of March, and the same modelling estimates that, by 23 March, between 1.9 and 2.3 million people had been infected. The chance to suppress transmission at a much earlier stage of the epidemic had been missed.

In analysing these events, what distinctive contribution can philosophy of science make? We are not a public inquiry; it is not our role to apportion blame or credit, to demand apologies, or to make recommendations that are specific to a single national context. We are not journalists; it is not our role to tell gripping narratives. We have not been at the table when decisions are made, so we cannot offer first-hand reflections and insights. But one useful thing we can do is study the table. We can analyse, from a philosophical point of view, the dynamics of the relationship between government and advisor, and that between science and values, in unprecedented and dire circumstances, in the hope of extracting generalizable lessons. That is my project here.

Why do this? I have three goals in view. One is to arrive at norms for effective scientific advising that may usefully generalize to other national contexts and to other major crises, including future pandemics. Another is to better understand how *normal* advising differs from advising *in extremis*, when the lives of a significant fraction of a country's citizens are in immediate peril and there is no adequate pre-existing plan or procedure for managing the risk. I will argue that there is a normative difference between these two contexts of scientific advising, and I aim to clarify the nature of the difference. A third is to better understand the relation between science and values, and in particular the role that non-epistemic (ethical, social, political) value judgements may legitimately play in scientific advising.

The process of scientific advising in the UK has been well documented, providing us with a rich set of resources on which to draw. SAGE ultimately met 74 times in 2020. Since late May 2020, the minutes have been made publicly available, usually within one month of the meeting. SAGE is represented on COBR (also known as Cobra), the UK’s primary decision-making body for civil contingencies, which is traditionally (but not always) chaired by the Prime Minister. Although minutes from COBR are classified, it is reasonable to assume that the SAGE minutes, along with other research papers and memos released by SAGE, reflect the scientific advice being provided to COBR at the time in question. A complication here is that, according to the Prime Minister’s Chief Adviser at this time, Dominic Cummings, COBR was often bypassed in practice, with real decision-making occurring during informal meetings in 10 Downing Street (HSCC/STC, 2021, Q973-Q979). I will nonetheless assume that the SAGE minutes provide a fair account of the scientific advice feeding in to these informal meetings.

NERVTAG formally advises the Department of Health and Social Care, but throughout 2020 has collaborated closely with SAGE and has significantly overlapping membership. This group met more than 40 times in 2020, and its minutes have also been made publicly available. SAGE also has two important subgroups: SPI-M–O (Scientific Pandemic Influenza Group on Modelling, usually known by its earlier name of SPI-M) and SPI-B (Scientific Pandemic Insights Group on Behaviour). While the minutes of these groups are not published, SPI-M-O has regularly produced "consensus statements" that are intended to convey to SAGE the consensus view within the group, and these are publicly available. A further source of evidence is public testimony to the Health and Social Care Select Committee and the Science and Technology Select Committee. I will be drawing here on all these sources, but with a special focus on the period January-March 2020.

The analysis that follows will be structured around two topics: (1) the distinct roles of “normatively heavy” and “normatively light” advice, and (2) the role of reasonable worst-case scenarios in strategic planning. In each case, I will draw on the available resources to highlight key features of the advisory process, leading me to propose generalizable norms for scientific advising in extremis. I will conclude with an epilogue that shifts the focus to September 2020 and considers whether my recommendations would have helped in that context.

## Who makes the value judgements? Normatively heavy and normatively light advice

The slogan “advisers advise and ministers decide”, coined by Margaret Thatcher in 1989, is a popular saying in UK government circles. The Chief Medical Officer during the pandemic, Chris Whitty, has himself used it to describe how he sees the relationship between SAGE and government (HSCC, 2020, Q646). But what does it mean in practice? Should scientific advisers limit themselves to advising on what means would be effective in relation to which ends, without endorsing any particular course of action? Or should they issue imperatives that ministers can either follow or ignore?

Whichever form their advice takes, I take it there will be at least *some* non-epistemic (ethical, social, political) value judgements involved in formulating the advice. For example, the judgement about which options to mention in one’s advice, and which to omit, already involves a value judgement (see the many cases discussed by Douglas (2009), Steele (2012), Elliott and Richard (2017)). However, some value judgements are more momentous than others. A value judgement is momentous to the extent that decisions turn on it—to the extent that it changes the course of the pandemic response. Not all value judgements are like this. For example, a decision about whether or not to highlight a statement in bold font is a value judgement, but one that is usually non-momentous. Advisers can seek to avoid making momentous value judgements by adopting more circumspect forms of advice.

It will be helpful, for my purposes, to introduce a broad distinction between *normatively light* and *normatively heavy* scientific advice. Normatively light advice *declines to endorse any particular course of action*, focussing instead on conditionals and means-end relationships. It may take the form “If your goal is this, then this would be an effective means. If, on the other hand, your goal is this…” or the form “If you do this, then we expect this to happen…”.

An influential member of SAGE at the time, Neil Ferguson (Head of the Imperial College COVID-19 Response Team), gave a clear statement of a “normatively light” conception of SAGE’s role in his testimony to the Science and Technology Committee in March 2020:

To be clear, SAGE does not recommend policy. SAGE makes judgments about science, looking at scientific evidence, including about how rapidly the epidemic is moving and what the likely lethality is, and not recommendations about interventions but insights into what interventions might have what effect. (STSC, 2020a, Q9).

Normatively light advice is not free of value judgements, so it would be inappropriate to call it “non-normative”. For example, judgements are made regarding which interventions are worthy of inclusion in the list of possibilities that are considered. However, the endorsement of any particular course of action is avoided, and the role of value judgements is minimized.

Normatively heavy advice takes the further step of *endorsing a course of action and (at least implicitly) recommending against alternative courses*. It may take the form: “Do one of these things” or simply “Do this.” Since a particular course of action is now being endorsed, there is an additional value judgement being made that is not present in normatively light advice.

This distinction is not sharp. As we will see, there are ways of formulating advice that blur the boundary between normatively light and normatively heavy. Nonetheless, the distinction is helpful for understanding what happened in the UK in early 2020. When we look at the evidence, we see it is not correct that SAGE’s advice was entirely normatively light. SAGE’s advice was mostly normatively light during January and February, with exceptions, before shifting to a normatively heavier approach on 5 March. There was then a major change around 16–18 March in the nature of the strategy SAGE endorsed.

## Types of advice: analysing the evidence

### The initial strategy

From the beginning, the SAGE minutes are in tension with Ferguson’s suggestion that SAGE “does not recommend policy”. At its 13 January extraordinary meeting, NERVTAG considered the idea of port-of-entry screening for symptoms, describing it as “not advised” (NERVTAG, 2020). In the context, to describe an action as “not advised” is to recommend against it. The SAGE minutes for 22 January affirm that “SAGE supports NERVTAG's position … on the value of port screening” (SAGE, 2020k). However, I see it as a reasonable generalization that SAGE’s advice is mostly normatively light until the end of February.

There is a draft paper, dated 26 February and discussed at a SAGE meeting on 27 February 2020, that I take to capture the consensus view of SAGE at that moment (SAGE, 2020b). The paper presents various mitigation options, including social distancing (where people are instructed to remain a specified distance apart) and shielding or “cocooning” (where clinically vulnerable people are instructed to self-isolate entirely), and various ways of combining them. The paper notes that "Implementing a subset of measures (e.g. the first three) would be expected to have a more moderate impact – still substantially reducing peak incidence, while making a second wave of infection in Autumn less likely. This might be the preferred outcome for the NHS." (SAGE, 2020b). The phrase “might be preferred” stops short of explicitly endorsing this course of action.

SAGE adds that “It is a political decision to consider whether it is preferable to enact stricter measures at first, lifting them gradually as required, or to start with fewer measures and add further measures if required" (SAGE, 2020b). This is striking because the decision in question is not *purely* political. The right decision depends *in part* on normative/evaluative considerations (How bad is it to compromise civil and economic liberties more than is necessary? How bad is it to expose citizens to more risk than necessary?), but it also depends in part on *epidemiological* facts about the comparative effectiveness of initially strict measures which are then relaxed as opposed to initially lax measures which are then tightened.

When a judgement inherently involves evaluative/normative considerations, and yet also depends sensitively on scientific facts, I will call it a *mixed judgement*.^4^ Pandemics are by no means the only cases in which mixed judgements arise. However, the stakes in such a case are unusually high. These cases involve momentous mixed judgements, where the judgement either way is likely to affect the lives of millions of people in profound ways. One advantage of avoiding explicit recommendations, from the point of view of an adviser, is that it is a way of avoiding momentous mixed judgements. The “It is a political decision…” line is an instance of SAGE deliberately avoiding a momentous mixed judgement and instead handing it over to political leaders.

An updated draft, dated 4 March, included a comment that more explicitly adopts a normatively light approach: “SAGE has not provided a recommendation of which interventions, or package of interventions, that Government may choose to apply” (SAGE, 2020c). In the early days of the crisis, SAGE has already seen modelling results clearly showing the potential for a public health catastrophe, and has already seen evidence of community transmission of COVID-19 in the UK, but (in line with the earlier quotation from Ferguson) they have not yet made explicit recommendations. However, the paper also notes that SAGE will, on 5 March, consider the “optimal combination of interventions”, the “optimal point to enact these interventions”, and the “duration that these interventions should be in place”.

The SAGE minutes for 5 March 2020 give us the results of these discussions. Here we find some very cautiously worded recommendations:

8. SAGE advised that the science supports a combination of case isolation and whole family isolation.

9. The science supports that a third intervention has epidemiological advantages: to socially isolate those in vulnerable groups (the elderly and those with underlying conditions) approximately 2 weeks after these initial interventions.

10. If implemented in combination as modelled, this set of measures is understood to most effectively delay and modify the epidemic peak, and reduce mortality. (SAGE, 2020i)

The phrase “the science supports” inhabits the borderline between normatively light and normatively heavy advice. It identifies a single course of action as “supported” without explicitly stating that it *ought* to be pursued. The most striking aspect of the recommendation to my eye is that *no other interventions* (such as social distancing, border closures, business closures, school closures, transport closures or mask wearing) are recommended at this time.

These recommendations were reflected in an updated draft paper dated 9 March and discussed on 10 March (SAGE, 2020d). The comment about “implementing a subset of measures” is now modified to include an explicit endorsement: "A combination of these measures is expected to have a greater impact: *implementing a subset of measures would be ideal*. Whilst this would have a more moderate impact it would be much less likely to result in a second wave” (SAGE, 2020d, italics added). In context, this amounts to recommending against implementing the *full* set of measures considered.

### Reasons for the initial strategy

What was the reasoning here? In these documents (the 5 March minutes, 9 March paper and the 10 March minutes), we see that SAGE made a controversial and fateful choice: the choice to advise against a course of action that might lead to a second wave later in the year by suppressing transmission too aggressively in the short term. What SAGE initially endorsed was *not* a strategy of maximally aggressive suppression: it endorsed implementation of a “subset” of the measures considered. The prevailing view at this time was that the costs of maximally aggressive suppression would exceed the benefits, because suppressing transmission completely would lead to a catastrophic, unmitigated epidemic when the measures were relaxed.

**Fig. 1:**
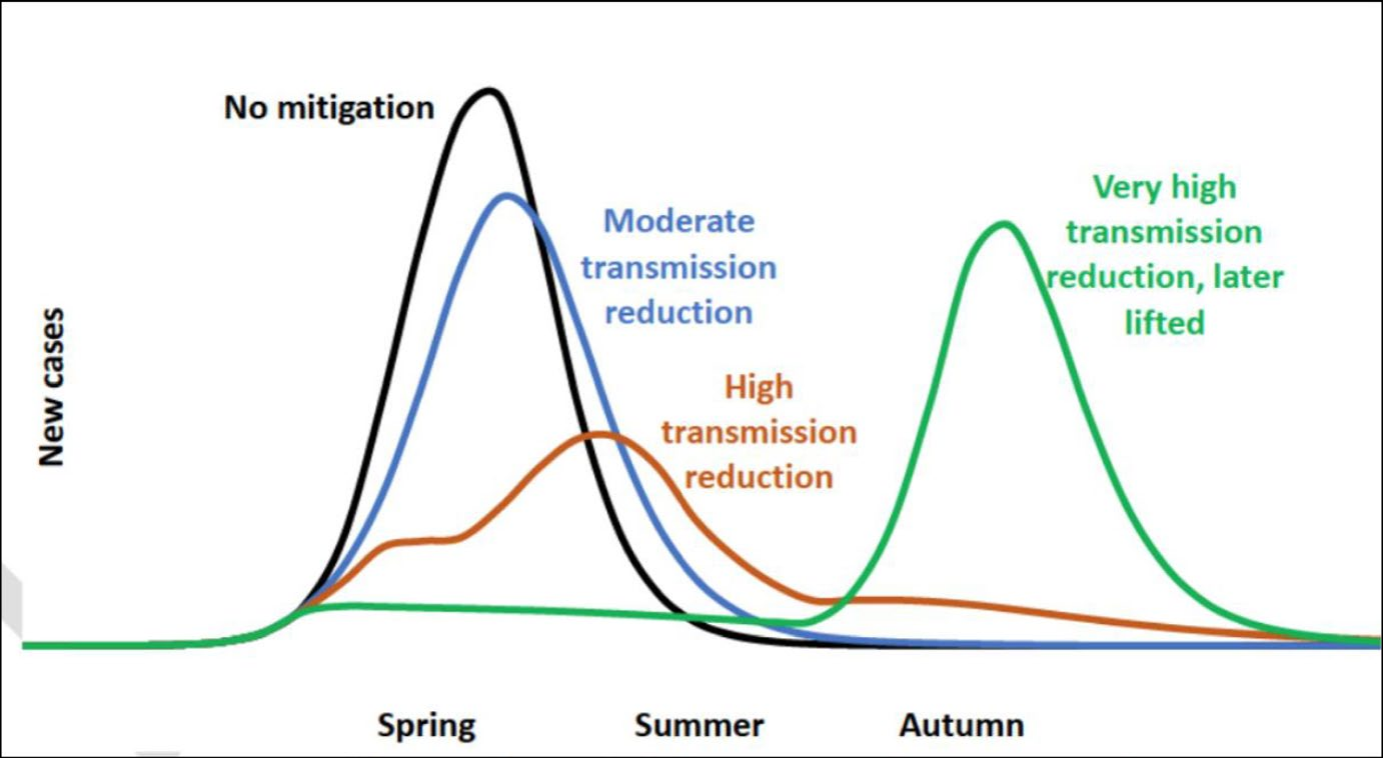
A sketch of a graph from SAGE (2020d), dated 9 March 2020. The “high transmission reduction” line depicts a strategy in which herd immunity is achieved by the summer through natural infection. The “very high transmission reduction” line depicts a strategy in which aggressive suppression in the spring leads to a disastrous wave of infection in the autumn, again leading to herd immunity through natural infection

This received wisdom is captured by a sketch of a graph in the 4 March and 9 March draft papers, which appears designed to illustrate the superiority of “high transmission reduction” over “very high transmission reduction, later lifted” (Fig. 1). At the time, SAGE was perceived by its critics to be following a “herd immunity strategy”, whereas the Chief Scientific Adviser, Sir Patrick Vallance, vehemently denied that there was any such strategy in private emails subsequently released to the BBC (Kermani, 2020). We can see that the recommended strategy between 5 and 16 March was a “high transmission reduction strategy” that aimed to flatten the curve without suppressing it completely. This is a strategy in which herd immunity is achieved and is the means by which a second peak is avoided. The resistance to the term “herd immunity strategy” is understandable, but nonetheless the achievement of herd immunity through widespread infection was a central component of the initial strategy.

To understand the underlying rationale for this advice, we need to understand a piece of conventional epidemiological wisdom: “flatter” epidemics spread out over a longer period are, in theory, less severe than fast epidemics with sharp peaks, both because the healthcare system is less overstretched at any given time and because the total number of people infected is likely to be lower.^5^ A second important point is that healthcare demand is normally much greater in the autumn and winter than in the summer, and SAGE believed peak healthcare demand would be reduced by guiding the peak towards summer. Vallance gave a rationale along these lines in his press briefing of 12 March (10 Downing Street, 2020), and SAGE member John Edmunds gave a similar rationale in a media interview on 13 March (Channel 4 News, 2020, 9:50–23:45).

Note that this line of thought only makes sense if we make a variety of pessimistic background assumptions, including that vaccines will not become available soon enough and that long-term behavioural changes will not be instilled. These pessimistic assumptions will be interrogated later, in Sects. 5 and 6.

### The sudden change of strategy

A major change of approach becomes evident in the published material on 16 March. On that day, SAGE discusses several new modelling papers, including one from the Imperial College COVID-19 Response Team (Ferguson et al., 2020). This paper explicitly contrasts mitigation strategies that aim *not* to suppress transmission completely (i.e. the type of strategy endorsed by SAGE in the 9 March paper) with a strategy of maximally aggressive suppression, including school closures, showing that the second type of strategy has the potential to lead to far fewer deaths. The paper contains the line: "We therefore conclude that epidemic suppression is the only viable strategy at the current time" (Ferguson et al., 2020).

For some time, models discussed by SAGE had been showing that, without maximally aggressive suppression, the demand on critical care beds would be enormous. SAGE had already written on 26 February that “In the reasonable worst-case scenario, demand on beds is likely to overtake supply well before the peak is reached” (SAGE, 2020b). This was not news, to SAGE, on 16 March. But prior to 16 March, the prevailing view nonetheless opposed maximally aggressive suppression, for the reasons noted above.

**Fig. 2:**
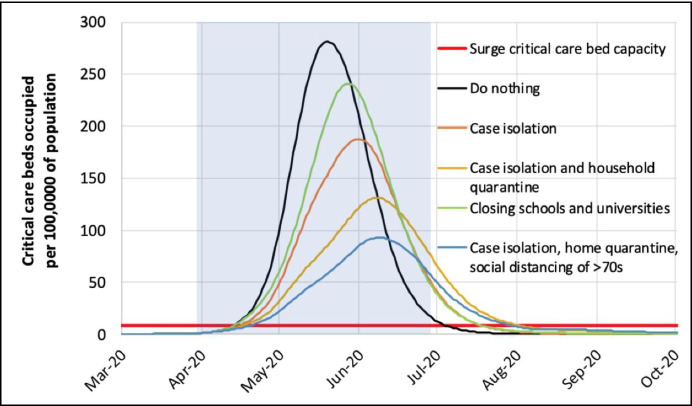
Projected demand for critical care and critical care capacity, from Ferguson et al. (2020). The projections indicate that, without aggressive suppression measures such as school closures, demand for critical care would exceed supply many times over

So what *was* the news on 16 March? For one thing, reliable data about critical care capacity was plotted on the same graph as “reasonable worst-case scenario” projections regarding demand for critical care, revealing the enormous size of the mismatch (Fig. 2). Even with mitigation measures in place, the Imperial model projected that demand, in a reasonable worst-case scenario, would exceed supply by at least a factor of eight. It is possible that other SAGE members, although well aware that “demand on beds is likely to overtake supply well before the peak is reached”, had not fully appreciated the size of the mismatch. A slide presented by Vallance in a public briefing on 12 March offered a much more optimistic view on which there was no mismatch at all (10 Downing Street, 2020, March). Nonetheless, I still find this an unconvincing explanation for the change of approach.

**Fig. 3:**
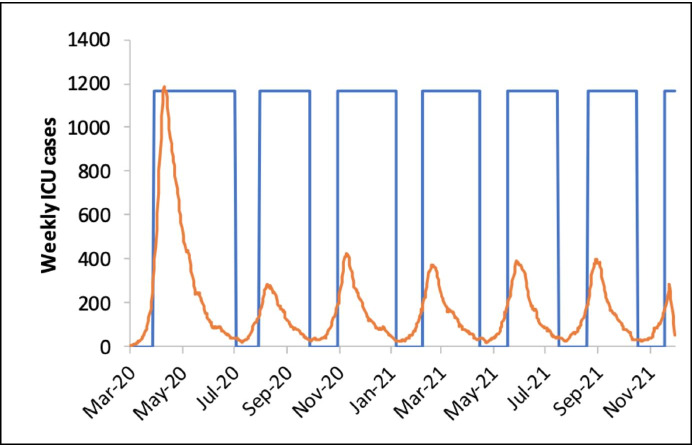
A graph from Ferguson et al. (2020), in which the blue-outlined blocks represent periods of school closure. This is a projection of the consequences of closing schools for about two thirds of the time, indefinitely, until a vaccine is developed

A better explanation, to my eye, appeals to the idea that *new policy options came into view*—options that had previously been seen as too radical to merit serious consideration. The strategy Ferguson et al. endorse involves not just *short-term* school closures, but *regular, sustained school closures* until a vaccine or effective treatment is implemented or until herd immunity is achieved. The projection is that school closures will be needed again whenever cases start to rise sharply, and that this will have to be done for roughly two thirds of the time for at least 18 months (Fig. 3).

Prior to 16 March, I can find no concrete evidence of this having been considered by SAGE as a realistic possibility. The expectation was that measures would be one-shot, time-limited (for a period of at least 8 and at most 13–17 weeks, depending on the measure), and that the epidemic would return when they were lifted. The new possibility, which Imperial now describes as the *only viable option*, involves a level of sustained school closure that was previously off the table. According to Cummings, an option of this type was discussed informally on the evening of 13 March (HSCC/STC, 2021, Q1003), but to my knowledge its first appearance in published official documents is in the Imperial paper dated 16 March.

The Ferguson et al. paper therefore made (in a rather discreet way) some truly momentous mixed judgements. There was a value judgement involved in regarding sustained school closure as a live option worth modelling at all, but the most significant value judgement was to regard that option as so clearly preferable to an overwhelmed healthcare system, given the model projections, that it was the *only viable option*, implying an urgent normative imperative to pursue it.

The Imperial paper directly informed the advice given by SPI-M–O to SAGE 16 March, in which SPI-M–O reported:

It was agreed that the addition of both general social distancing and school closures to case isolation, household isolation and social distancing of vulnerable groups would be likely to control the epidemic when kept in place for a long period. *SPI-M-O agreed that this strategy should be followed as soon as practical, at least in the first instance*. (SPI-M-O, 2020b, italics added)

This is another example of normatively heavy advice. It is an endorsement of Ferguson and colleagues’ recommendation.

SAGE itself, at this moment, continued to prefer more circumspect advice. The minutes of the 16 March meeting record that “SAGE advises that there is clear evidence to support *additional social distancing measures* be introduced as soon as possible." (SAGE, 2020e, italics added). The nature of the additional measures was left open, and school closures were not yet recommended. Two days later, this changed. On 18 March, SAGE advised that “available evidence now supports implementing school closures on a national level, as soon as practicable to prevent NHS intensive care capacity being exceeded” (SAGE, 2020l).

As on 5 March, the language of what the science or evidence “supports” leaves some room for debate about whether this was an explicit recommendation, since it does not explicitly say what the government ought to do or should do. Nonetheless, it is clear in the context that a specific course of action was endorsed. The recommendation was followed, and schools closed on 20 March.

## Types of advice: reflections and a proposal

What can we learn from this case? My interest here is in generalizable norms for scientific advising in extremis. When I say “in extremis”, I mean that the situation is one in which the lives of a significant fraction of a country's citizens are in immediate peril and no pre-existing plan or procedure exists for managing the risk.^6^ Given the way I have defined “in extremis”, it is clear that, *ideally*, countries will never find themselves in extremis, because they will have pre-agreed plans and procedures for managing major risks. So our project here is one of constructing norms for effective scientific advising in a far-from-ideal situation.

One important reference point for this type of problem is Michael Walzer’s work on the “problem of dirty hands” (Walzer, 1973, 1977, 2004). Walzer argues that there is a normative difference between political leadership in normal times and political leadership in extremis, or, to use his preferred term, “supreme emergency”. In normal times, a political leader should adhere to the ordinary moral norms of the community they lead. But in extremis, when the moral community itself is in immediate peril, those norms can be permissibly overridden. A political leader may violate the ordinary moral norms of the community in order to avoid catastrophe. For example, perhaps a leader could permissibly divert enemy bombing away from strategically important city centres on to working class residential areas, as the UK did in World War Two (Burri, 2020).

I want to make a Walzer-inspired point in relation to scientific advising. I suggest that the norms of scientific advising in normal times, though present for good reasons, can sometimes be permissibly overridden in extremis. In particular: there are good reasons for advisers to avoid normatively heavy advice in normal times, but in extremis this type of advice can be appropriate—and I contend that the “aggressive suppression now” recommendation of 16–18 March is an example of normatively heavy yet appropriate advice.

Why is normatively heavy scientific advice inappropriate in normal times? It is inappropriate because it violates norms of accountability. A decision is democratically accountable if the person who made it can be held to account for it by the public. Holding a decision-maker to account can take many forms, but typically involves compelling them to give a public account of the reasons for their decisions and to respond to challenges, and it also involves the threat of democratic consequences (such as electoral defeat or removal from office) if their account reveals a mismatch between their reasons and the values of the voters. Accountability is widely taken to be an important norm of decision-making in democratic societies.^7^

A structure in which advisers make a single, normatively heavy recommendation, to be followed or rejected by decision-makers, leads to a problem of accountability. In making normatively heavy advice, scientific advisers claim for themselves the ability to make momentous mixed judgements that include a substantial evaluative component, such as the judgement that healthcare system collapse is normatively worse than prolonged school closures. The advisers are not representatives and are not subject to the possibility of democratic consequences, and there is no particular reason to think the advisers’ value judgements will reflect the values of voters. This leads to the concern that there is insufficient accountability for these momentous judgements.

Ministers, meanwhile, having in effect outsourced the task of weighing conflicting values (such as the value of public health and education) to their advisers, find themselves in a position in which the only public rationale they can give for their decisions is: “I trusted my advisers and accepted their recommendation” (or simply, “I followed the science”). The ministers can be praised or blamed for trusting their advisers, but the underlying normative reasons and values that led to the decision will not be properly accounted for or subjected to democratic acceptance or rejection.

We can describe the resultant situation as an *accountability gap*: no one is properly democratically accountable for value judgements that shape people’s lives in dramatic and long-lasting ways. In such a situation, there is the potential for policy responses to emergencies to float free of what a fully informed citizenry would actually support, if the decisions were put directly to them.

I see this concern about accountability as a serious one, and one that would probably win the day in normal times. In normal times, it provides a good reason for scientific advisers to limit themselves to normatively light advice in which no specific policy option is endorsed. Yet I do not see this as a necessarily decisive reason to avoid normatively heavy advice, and I think it does not always win the day in extremis.

What is different about advice in extremis? A piece of scientific advice can be considered abstractly as a signal sent by an adviser to a decision-maker that influences their actions. As with any signalling system, the sender does not have full control over how the receiver will react. Their signal merely shifts the probabilities of different actions. Moreover, there will be noise in the channel—misunderstandings are possible. In this context, different types of signal are not all on a par in terms of their influence. A clear imperative signal–*Do X now!*—is much more likely to produce a specific action *X* than a complex description of possible actions and their likely consequences. In normal times, I take it that the imperative to avoid accountability gaps is generally more important than the imperative to elicit a specific policy response. In extremis, this situation can sometimes be reversed.

To put the point more precisely, my contention is that there can be circumstances in which:

- An adviser reaches the justified conclusion that a specific action, *X*, is the best available way to avert a normatively terrible outcome, *O*;
- Providing political leaders with a normatively light presentation of various alternatives, with* X* among them, would not reliably lead to *X* being implemented;
- *O* would be sufficiently catastrophic for the whole of society that the imperative to reliably bring about *X* overrides any other obligations the adviser normally has, such as the obligation to facilitate democratically accountable decision-making.

In such a situation, I contend that the adviser faces an analogue of the type of situation Walzer described as a “dirty hands” situation. Their normal obligations are overridden by an exceptionally pressing need to elicit a specific policy response. I also contend that the advisers who first realized, on or around 16 March 2020, that immediate school closures and other strong measures (such as general social distancing) were the only credible means of averting an overwhelmed healthcare system were in the type of situation just described.

One might agree that the general type of situation characterised by (a)–(c) could happen and yet disagree that the events of mid-March are a genuine instance of it. Critics of the Ferguson et al. model have argued that, due to the tentative nature of its modelling assumptions, it did not generate a normatively adequate justification for its recommendation (Northcott, 2021; Winsberg et al., 2020; but see also the response from van Basshuysen and White, 2021). Importantly, though, we should avoid overstating the significance to the case of this one model. As we have seen, SAGE had held since late February that “in a reasonable worst-case scenario, demand on beds is likely to overtake supply well before the peak is reached”. Many different models, plus news reports from Lombardy and Wuhan, supported this claim. In the context, the main contribution of the 16 March Ferguson et al. model was to bring into view a policy option (“suppression”) that, in the model, averts that outcome by means of indefinitely sustained, repeated periods of school closure.

The normative justification for this policy option was that it *could realistically* avert the disaster that a reasonable worst-case scenario would (as a matter of consensus) bring, and it was the *only* live policy option that could do so. The role of the model within the wider case was to show the insufficiency, given a set of intentionally pessimistic planning assumptions, of less interventionist approaches and the potential adequacy of a set of interventions including school closures and general social distancing.

If we grant that the advisers on SPI-M-O were indeed justified in concluding, on 16 March, that a suppression strategy was justified, how should they have communicated their advice? In normal times, they should have tried to enable accountability by laying out different options for decision-makers along with projections of their likely consequences, without endorsement. But this was a situation of grave emergency in which conditions (b) and (c) were also satisfied. There was no strong reason to think decision-makers would reliably make the same mixed judgements given the same modelling and data. Misunderstandings were likely. There was some reason to suspect decision-makers would resist a dramatic, sudden change of strategy—since any political leader will be averse to U-turns that undermine public confidence in their grip of the situation. And the situation was sufficiently dire to override norms of accountability, since hundreds of thousands of lives were in the balance.

That leads me to my first proposal: I suggest that the norm against accountability gaps, although reasonable in normal times, can sometimes be appropriately suspended in extremis. Some accountability gaps may be tolerated*. Scientific advisers may permissibly make normatively heavy recommendations, including simple, unambiguous imperatives to implement a specific policy option, where they have a justified belief that this option is the only robust way to avert a catastrophic outcome.*

One might object that SAGE was rather *too* willing to endorse a specific policy option on 5 March, namely the “high transmission reduction” option—believing it, at the time, to be the only available means of averting an even worse disaster than short-term healthcare system collapse. Had this belief been justified (i.e. condition a), then according to my proposal they were justified in making that recommendation—since conditions (b) and (c) were plausibly also satisfied. This may lead to some unease about my proposal. However, I do not think their belief was justified. It was the result of an impoverished set of policy options, based on the constraints imposed by a particular set of planning assumptions. Those assumptions will be the focus of the next section.

## Reasonable worst-case scenarios: analysing the evidence

I have sometimes heard it suggested that SAGE’s approach to the pandemic was not “precautionary” enough—not sufficiently concerned with avoiding the worst-case outcome. The SAGE minutes show this to be too simplistic. As we have seen, a concern with preventing what they saw as a reasonable worst-case outcome, in which excessive intervention in the spring led to a disastrous second wave in the autumn, was the guiding principle behind SAGE’s advice during an important period in late February and early March. In this section, I want to zoom in on the role played by this reasoning about the “reasonable worst-case scenario” (RWCS).

### The concept of a reasonable worst-case scenario (RWCS)

The concept of a “reasonable worst-case scenario” (RWCS) has been at the core of SAGE’s approach to the pandemic from the beginning. An RWCS is a set of assumptions that reflect one way in which the epidemic in the UK may unfold. The set of assumptions is “reasonable” in the sense of being regarded by SAGE as a serious possibility. It is “worst-case” in the sense of being at the pessimistic end of the range of serious possibilities.

The RWCS concept has been prominent in civil contingencies planning in the UK since at least 2009, when it played an important role in the response to the H1N1 (“swine flu”) pandemic. A succinct explanation of the concept was presented by SAGE to the Science and Technology Select Committee in 2010:

The reasonable worst case is a concept developed for emergency planning in the UK. This concept is designed to exclude theoretically possible scenarios, which have so little probability of occurring that planning for them would lead to a disproportionate use of resources. They are not predictions of what will happen but of the worst that might realistically happen, and therefore we would expect most pandemics to be less severe and less widespread than the reasonable worst case. By planning for the reasonable worst case planners are assured that they have a high probability of meeting the demands posed by the hazard should it occur. (STSC, 2010, Appendix A)

There are two broad rationales for taking a particular interest in the RWCS. One is the idea that, *if you plan for the RWCS, you will also be well prepared for less severe scenarios*. Planning for the reasonable worst-case is thus assumed to be a way of robustly preparing for a wide range of scenarios. The other rationale is the idea that, in a situation of uncertainty, giving special weight to the RWCS in decision-making is normatively justified by an *imperative to avoid the worst possible outcomes*.^8^ The above paragraph suggests the first rationale has traditionally driven SAGE’s interest in the RWCS. However, the recommendations made by SAGE, and the importance to those recommendations of avoiding a disastrous second wave in the autumn, suggest that the second role also played at least an implicit role in their deliberations.

### The initial RWCS

What was the RWCS, in the UK’s initial response to COVID-19? It was, in some respects, highly pessimistic. SAGE’s RWCS planning assumptions, adapted from an existing RWCS for pandemic influenza, set out a scenario in which 80% of the population is infected over a period of about nine weeks, with 50% displaying symptoms, and an infection fatality rate (IFR) of 1%. The result would have been around 520,000 excess deaths within three months. In a draft dated 4 March 2020 (SAGE, 2020n), it was also explicitly assumed that no effective treatments or vaccines will become available either before or during the epidemic. This line was deleted from the version dated 6 March (SAGE, 2020o), but seemed to tacitly guide strategic planning.

SAGE also made some highly pessimistic background assumptions that, although not formally part of the RWCS planning assumptions, are important for understanding the advice given at this time. First, SAGE was initially pessimistic about the prospects for mass testing or contact tracing (where the contacts of infected individuals are tracked down and instructed to self-isolate). The minutes of 18 February state that “when there is sustained transmission in the UK, contact tracing will no longer be useful” (SAGE, 2020j). Second, SAGE initially assumed that interventions (such as social distancing and shielding or “cocooning”) would be one-shot interventions for a period of 13–17 weeks. Some modelling was done of a scenario in which they are sustained for 26 weeks, but 13–17 weeks was the assumption in SAGE’s written advice. Third, SAGE assumed partial compliance. For example, it assumed that 50% would comply with household quarantine. SAGE described this 50% level of compliance as “high levels of compliance” and warned that such a level of compliance “may be unachievable in the UK population” (SAGE, 2020c).

There was also pessimism about the ability of the healthcare system to cope with demand in the RWCS, regardless of the interventions taken. There is a line in SAGE’s 26 February draft advice that says “in the event of a severe epidemic, without action, the NHS will be unable to meet all demands placed on it. In the reasonable worst case scenario, demand for beds is likely to overtake supply well before the peak is reached” (SAGE, 2020b). The words “without action” were deleted between the 26 February draft and the 3 March draft (SAGE, 2020c), presumably to take account of the new modelling that had become available on 2 March. The new modelling suggested that “without action” was misleading, since none of the actions being considered at that time would be enough to prevent demand for healthcare outstripping supply.

Yet in other respects, the RWCS assumptions were surprisingly optimistic. SAGE (in the 4 March draft) assumed that R_0_, the basic reproductive rate of the virus in the absence of mitigation, was 2.4, leading to a doubling time of 4–6 days. Estimates varied a great deal at the time, and still do, but this was, even then, towards the lower end of serious estimates for R_0_. A study published in *The Lancet* on 31 January had estimated R_0_ at 2.7 (Wu et al., 2020). On 11 February, researchers at the Theoretical Biology and Biophysics unit at the Los Alamos National Laboratory, USA, released a preprint estimating that R_0_ was between 4.7 and 6.6 (Sanche et al., 2020). SAGE’s line about R_0_ was deleted in the 6 March draft, in which no estimate of R_0_ was included.

There was also another optimistic background assumption, introduced on 25 February, that surveillance “should provide evidence of an epidemic around 9–11 weeks before its peak” (SAGE, 2020g). Just as R_0_ being higher than expected was not part of the RWCS, surveillance being poorer than expected was also not part of the RWCS. In short, the RWCS assumptions were a mix of bleak pessimism and excessive optimism.

### Consequences

What were the consequences of these choices? The costly delays between 2 and 23 March can be seen as consequences of planning assumptions made around the beginning of this period. The combination of an optimistic estimate for R_0_, optimism about surveillance, and an assumption that 13–17 weeks was the realistic maximum duration for interventions, made it seem optimal to delay the introduction of social-behavioural interventions that were in fact urgently needed.

This problem was compounded by continued scepticism, up to at least 16 March, about the idea of maximally aggressive suppression. As we have seen, between 5 and 16 March, SAGE’s advice was explicitly *against* maximally aggressive suppression and in favour of more moderate measures. This initially sceptical attitude towards maximally aggressive suppression can also be traced to the RWCS planning assumptions. In a reasonable worst-case, no effective treatment, contact tracing system or vaccine becomes available before measures are relaxed, and no long-term behavioural changes are instilled, so total suppression leads inevitably to the epidemic returning with unmitigated force in the autumn, infecting 80% of the population and overwhelming the health service. This corresponds to the “very high transmission reduction” line in Fig. 1. In that bleak scenario, we come to bitterly regret the aggressive measures adopted in the spring.

A defender of SAGE’s initial strategy might object: the UK did indeed endure a dreadful wave (or pair of waves, if we count the emergence of the Alpha variant as a new wave) in the autumn/winter of 2020–21, just as the “very high transmission reduction” line projects. On 1 September 2020, the official death toll (based on deaths within 28 days of a positive COVID-19 test) stood at 41,650. By 1 March 2021, it had risen to 124,288 (UK Government, 2021a). So, can we be sure the initial strategy was a mistake?

Note, however, that the autumn/winter epidemic was far from sufficient to achieve herd immunity through natural infection. This can be clearly seen from the emergence of a new wave (i.e. a third or fourth wave, depending on how one counts waves) in summer 2021, despite a large-scale vaccination programme (UK Government, 2021b). Had the epidemic been allowed to grow unsuppressed to the level of natural herd immunity without vaccination, as per the initial strategy, it would have grown much larger than the actual epidemic of autumn–winter 2021. Since the logic of the initial strategy was to tolerate a vast wave in the summer to prevent an even worse one the following winter, the smaller wave that actually occurred does not provide a retrospective vindication of the initial strategy. It is never easy to evaluate counterfactuals, but I think we have good evidence that the initial strategy would have led to disastrous results, had it not been abandoned.

## Reasonable worst-case scenarios: reflections and proposals

Let us return to one of the underlying principles apparently guiding the use of RWCSs: if you assume you are in the RWCS, and plan accordingly, then you will be as well prepared as possible for less severe scenarios. I will call this the *RWCS principle*. This case, I suggest, shows us some important exceptions to the RWCS principle.

One problem with the RWCS principle arises when the RWCS is pessimistic in *most* relevant respects but not *all* of them. I will introduce the term “globally pessimistic scenario” for a scenario that is at the pessimistic end of scientific opinion regarding *all* of its planning assumptions, not just some. To be clear, these assumptions should still be at the population level, and may still include estimates of statistical variables. Clearly, we should not think: any individual *could* die from COVID-19, so the globally pessimistic scenario should include a 100% fatality rate. Instead, we should take the most pessimistic but still scientifically serious estimates of each statistical variable. SAGE’s RWCS was not globally pessimistic in this sense. It was in some respects optimistic. When the RWCS is not globally pessimistic, there is a serious risk that reality will be worse than the RWCS in those specific respects. This is what happened in relation to R, which was almost certainly above 2.4 before the spring lockdown in England, according to retrospective modelling, despite substantial attempts at mitigation (Birrell et al., 2021).

This leads me to a proposal: *the globally pessimistic scenario should be used in preference to an RWCS when what is desired is a general indication of how bad things could, in principle, become.* However, the nature of this role calls for further reflection, since it would be clearly unwise to let the globally pessimistic scenario dominate strategic planning. After all, the globally pessimistic scenario may well be extremely unlikely. To illustrate: the globally pessimistic scenario would be one in which compliance with behavioural interventions is poor (well below 50%), so allowing the globally pessimistic scenario to dominate planning would lead to unjustified scepticism about such interventions. There will be some contexts in which being aware of the globally pessimistic scenario may well be useful for specific decisions (e.g. when deciding how much of a given item of personal protective equipment to order, or when deciding how large to make emergency hospital wards) but it would be a clear mistake to premise all planning on a very low probability scenario.

A different problem with the RWCS principle is that there can be circumstances in which assuming you are in an RWCS justifies actions, delays, or omissions that will impair your response significantly if you are in a less severe scenario. For example, it makes sense to say that, *in a reasonable worst case*, no effective treatment or vaccine will become available, contact tracing will never become effective, and no long-term behavioural changes will be instilled, even if you delay the epidemic by several months. In this case, maximally aggressive suppression of transmission is likely to make things worse in the long run, as vividly shown in Fig. 1. It is better not to try it. But suppose you are in fact in a less severe scenario, in which one of these three pessimistic assumptions is false. In such a scenario, maximally aggressive suppression is likely to be far superior to more moderate action, in terms of both its public health consequences and its long-term economic consequences. So, if you plan for the RWCS, and adopt a strategy that involves aiming for the “high transmission reduction” line in Fig. 1, you in fact commit to a strategy that could lead to serious regret, if it subsequently transpires that vaccines, long-term behavioural change and/or contact tracing are more feasible than initially assumed.

To be clear, this second problem does not stem from the excessive optimism of some elements of the RWCS. Had SAGE’s planning scenario been globally pessimistic, this problem would still have arisen. It arises from over-reliance on a single set of planning assumptions. The basic problem is that, in a pandemic, less severe scenarios are not just scenarios in which all the same measures are needed, but a little less urgently or to a lesser degree. They can be scenarios in which a qualitatively different strategy is appropriate.

This leads me to another proposal concerning the use of RWCSs. Neither globally pessimistic scenarios nor RWCSs should be allowed to dominate strategic planning. For strategic planning, it is important to consider a *wide range of possible scenarios* and to *avoid reliance on the principle that preparing for the RWCS will avert disaster in less severe scenarios*. The apparent inevitability of a large wave as soon as measures were relaxed was sensitive to a specific set of worst-case planning assumptions, which assumed that measures could not be sustained until an effective treatment, vaccine, or contact tracing system was implemented. These assumptions dominated strategic planning up to 16 March. As soon as modellers relaxed one of those pessimistic assumptions and made room for hope of a vaccine, as in Ferguson et al. (2020), the strategic picture changed.

It might be objected that an imperative to consider a wide range of possible scenarios, including more optimistic ones, makes more sense in normal times than in extremis. Would considering a wider range of scenarios not simply have led to even more delays, and potentially to unfounded optimism? I accept that the scientific advisers were not in a position to assign precise probabilities or values to different scenarios, and were therefore not in a position to do an expected utility calculation or cost–benefit analysis. However, I do not think an inability to assign probabilities removes the obligation to consider a wide range of scenarios.

Even without assigning probabilities to scenarios, we can look for policies that deliver acceptable outcomes *robustly* (under a wide range of parameter values), as opposed to policies that deliver acceptable outcomes only under very specific circumstances. One natural suggestion, then, is that advisers should only recommend actions that robustly lead to acceptable outcomes across all realistic scenarios (see e.g. Bradley & Bright, 2020; Bradley & Roussos, 2021). Yet this may be asking too much in this context. From SAGE’s point of view, there was a wide range of scenarios in which maximally aggressive suppression leads to an unacceptable outcome, i.e. a devastating epidemic when measures were relaxed. This happens robustly in models in which there is no place for a vaccine, an effective treatment, effective contact tracing, or long-lasting behavioural change. The problem is not that this result was particularly fragile with respect to the variables *actually being modelled*, but that it was dependent on potential *game-changers* (i.e. the possibility of a vaccine, effective treatment or effective contact tracing becoming available and/or long-lasting behavioural changes being instilled) that were not modelled at all—and could not realistically be modelled within the approaches being used except as sudden, exogenous changes to the infection fatality rate (IFR) or reproductive rate (R) of the virus.

It may be tempting to say: in that case, what is needed is robustness across a wide range of models that *includes* potential game-changers. The problem with this suggestion is that game-changers can cut both ways. There are also scenarios in which the IFR or R rate is suddenly *increased* by new variants of the virus evolving, and in some of those unfortunate scenarios we might regret our failure to build up a degree of herd immunity when we had the chance.^9^ There is no principled reason for including positive game-changers in the model while excluding negative game-changers.

There is a deep problem here. Robustness across game-changer-free scenarios is not enough in a pandemic, but asking for policies that are robustly acceptable across all game-changer scenarios is asking the impossible. A choice has to be made as to which game-changers we want our planning to be robust against and which we do not, and here too it seems that momentous mixed judgements are unavoidable. In keeping with the conclusions of Sect. 4, I take it to be permissible for advisers to make these judgements: in extremis, we can tolerate the accountability problem this creates. However, these judgements, when made by advisers, should be *communicated* as clearly as possible to decision-makers so that they can be challenged if necessary.

This leads me to a fourth proposal: *when making a normatively heavy recommendation, scientific advisers should highlight, as part of their advice, the scenarios in which their recommended actions would lead to serious regret. For example, they should communicate which potential game-changers they have chosen to set aside when looking for robustly acceptable policies.*

For example, a recommendation to close schools should highlight that, if the fraction of asymptomatic infections turns out to be very high, such that herd immunity has already been reached, children would suffer a great harm for no public health benefit. Such warnings should then be contextualized, with (for example) an explanation of why betting on the fraction of asymptomatic infections being so high would be an extraordinary bet. Likewise, when SAGE recommended a “high transmission reduction” strategy over maximally aggressive suppression, it should have highlighted, in a contextualized way, the potential for this strategy to lead to serious regret—in the form of tens of thousands of excess deaths which could in retrospect have been prevented by pursuing a more aggressive suppression strategy at an earlier stage. Political leaders who are given a clear recommendation, together with a contextualized explanation of scenarios in which the recommendation would lead to serious regret, can make a decision about whether the risk of serious regret is acceptable to them or not.

## Summary of proposals

Before the epilogue, let us pause to review the proposals of the preceding sections:

1. Scientific advisers may permissibly make normatively heavy recommendations, including simple, unambiguous imperatives to implement a specific policy option, where they have a justified belief that this option is the only robust way to avert a catastrophic outcome.
2. The globally pessimistic scenario (a scenario at the pessimistic end of scientific opinion regarding *all* of its assumptions, not just some) should be used in preference to a reasonable worst-case scenario (RWCS) when what is desired is a general indication of how bad things could, in principle, become.
3. But neither the globally pessimistic scenario nor the RWCS should dominate strategic planning. Strategic planning should consider a wide range of possible scenarios and look for policies that lead to robustly acceptable outcomes, without relying on the (dangerous) assumption that policies which are optimal in the RWCS will successfully avoid disastrous outcomes in less severe scenarios.
4. When making a normatively heavy recommendation, scientific advisers should communicate (with context) information about scenarios in which acting on their recommendation would lead to serious regret. For example, they should communicate which potential game-changers they have chosen to set aside when looking for robustly acceptable policies.

The proposals are intended to be at the right level of generality to be, I hope, relevant to the management of future crises. To evaluate whether they do generalize in a useful way, I want to move forward in time—to September 2020.

## Epilogue: September 2020

In the autumn of 2020 and the subsequent winter, the UK experienced a second major epidemic. One could argue that the virus never went away, but there was a marked lull in the summer months, followed by a resurgence. This gives us a chance to revisit the themes of this article—the normative force of advice and the role of reasonable worst-case scenarios—in a new context.

Let us first consider the normative force of advice. On 21 September, as cases rose, SAGE offered the government a “shortlist” of non-pharmaceutical interventions (SAGE, 2020a). The shortlist consisted of “a circuit-breaker (short period of lockdown) to return incidence to low levels”; “advice to work from home for all those that can”; “banning all contact within the home with members of other households (except members of a support bubble)”; “closure of all bars, restaurants, cafes, indoor gyms, and personal services (e.g. hairdressers)”; and “university and college teaching to be online unless face-to-face teaching is absolutely essential” (SAGE, 2020a). SAGE commented that “a package of interventions will need to be adopted to reverse this exponential rise in cases” and added that “a consistent package of measures should be adopted which do not promote, or appear to promote, contradictory goals” (SAGE, 2020a). I see this advice as a compromise between normatively light and normatively heavy: a borderline case. A very general course of action is recommended, but options are deliberately left open, leaving many substantially different ways to follow the recommendation.

A single item from the shortlist (advice to work from home) was in fact implemented (see Cabinet Office, 2020). A package of measures was put together, but none of the other elements of the package were drawn from SAGE’s shortlist, and they were all clearly less radical interventions (e.g. attendance at weddings was limited to 15 people, down from 30). So, the government did indeed implement “a consistent package”, but the consistent package largely overlooked SAGE’s suggestions, and did not succeed in bringing R below 1.

I contend that a single, unambiguous recommendation would have been permissible here, for the same reason it was appropriate on 16 March: the situation was again one of grave emergency, and conditions (a)–(c) of Sect. 4 were again satisfied. Recommending the immediate implementation of all the measures on the shortlist would have left the government with a simple choice: implement the recommended measures, or be seen to manifestly ignore its own scientific advisers. This would have made implementation of sufficient measures more likely. By framing their advice in a disjunctive way, SAGE made it easier for the government to evade this choice by implementing a “consistent package” that was, foreseeably, insufficient to bring R below 1.

Meanwhile, RWCSs have continued to play a major role in planning. A new RWCS was drawn up in the summer and was confidential until leaked to *The Spectator* on 29 October and subsequently officially released (SAGE, 2020m). The new RWCS was not globally pessimistic, and was in some respects strikingly optimistic. In particular:

The scenario models incidence continuing as per current trends until the end of July 2020, with all non-household contacts assumed to be constant with current levels. Incidence is then assumed to double once by the end of August 2020, and double again during the first two weeks of September. *At this point, social contacts are reduced that reduce R to approximately 1, keeping infection levels steady until the end of October.* Two-week doubling times return throughout November (i.e. incidence quadruples through November), after which policy measures are put in place to reduce non-household contacts to half of their normal pre-March 2020 lockdown levels, while all schools contacts are assumed to be maintained. These measures are sustained until the end of March 2021. (SAGE, 2020m, italics added)

In other words, the RWCS assumes that unspecified but highly effective measures will be taken by the government in mid-September to bring R to approximately 1. It is remarkable to see such an assumption feature in a reasonable *worst-case* scenario. Did scientific advisers really regard this as a reasonable worst-case?

It seems that advisers had significantly less independence from other areas of government when constructing the new RWCS, in comparison with the first RWCS. In the spring, SAGE had set the assumptions of the RWCS. In the summer, by contrast, the RWCS was the result of negotiation with ministers:

This profile of increasing incidence to the end of November 2020, was agreed by SPI-M-O co-chair in collaboration with SAGE and Cabinet Office Civil Contingencies Secretariat and COVID-19 Taskforce. No specific assumptions as to what these measures may be were made. (SAGE, 2020m)

This comment leaves the role of ministers rather opaque. This is partly because the command structure at this time was also somewhat opaque. In place of COBR, two new committees, COVID-S and COVID-O, were created, with responsibility for decisions regarding strategic and operational planning, respectively (this is explained by the Secretary of State for Health in STSC, 2020b). I take it that at least one of these new committees was involved in agreeing the RWCS, and a SPI-M–O consensus statement from 16 September notes that “the RWCS *agreed with ministers* assumed that policy interventions would be made in mid-September to halt the rise in infections” (SPI-M–O, 2020b, italics added).

On 3 November, Vallance explained the process to the Science and Technology Select Committee in a way that seemed to further marginalize the role of scientific expertise: “We model what the Civil Contingencies Secretariat sees as a reasonable worst case and that is then modelled by the SPI-M modellers” (STSC, 2020c, Q1510).

So the government once again adopted a RWCS that was excessively optimistic in some respects—specifically, it was excessively optimistic about the government’s own actions. Effective measures were *not* taken in mid-September and cases continued to rise throughout October, leading to a national lockdown at the end of October. Although preferable to an unmitigated epidemic, this was a tragic outcome: the immense amount of resources invested in tracking early warning signs of a major epidemic, via an immense mass testing operation, did not translate into a swift and effective policy response when those warning signs were observed.

This highlights a different potential pitfall of RWCS-centred planning. An effective pandemic response can be hindered if planning assumptions are negotiated between scientific advisers and political actors, with political actors influencing the projected values of fundamental epidemiological parameters, such as *R*. *Clear limits need to be set regarding which aspects of modelling political actors can and cannot influence.*

To be clear, the lesson is not that policy-makers should have no input *at all*. What is needed is a clear framework for separating reasonable input (e.g. a description of the interventions they are willing to implement) from unreasonable input (e.g. an assertion that the unspecified interventions, whatever they may be, will have a particular epidemiological effect). It is not appropriate for political leaders to insist that planning assumptions for a “worst case” build in optimistic assumptions about their own actions.

## Conclusion

I see the case of September 2020 as providing further support for some of the proposals I put forward earlier in relation to January-March 2020. The events of September reinforce the point that situations of grave emergency may call for clear, direct recommendations from advisers that are not overly disjunctive. They also underline the danger of relying too heavily on a single set of planning assumptions which, while pessimistic in many respects, may also be too optimistic in some respects. My hope is that the proposals summarised in Sect. 7 can generalize to other pandemics, and other major crises, and that, if enacted, they would lead to better advisory and decision-making processes.

## Footnotes

1 In analysing this case, I have been particularly influenced by analyses of the interplay of science and values in other cases by Douglas (2009), Steele (2012), John (2015), Lewens (2019) and the contributors to Elliott and Richards (2017). Atkinson et al. (2020) have analysed the same events using different evidence, namely interviews with panels of witnesses, and highlight some different issues: the appropriate mix of expertise, and the choice between centralized and decentralized responses. Cairney (2020, 2021) has analysed the same sequence of events from a political science perspective. To keep the present paper focused and concise, I will not include comparisons between my reflections and those of others.

2 For a good narrative account of the period under discussion here, see Grey and MacAskill (2020).

3 “It is not possible for the UK to accelerate diagnostic capability to include Covid-19 alongside regular flu testing in time for the onset of winter flu season 2020–21” (SAGE, 2020f).

4 This term is inspired by Plutynski’s (2017) discussion of mixed judgements in cancer screening and Alexandrova’s (2018) discussion of mixed claims in the science of well-being.

5 See Handel et al. (2007) for an explanation of this second reason. To explain intuitively: once a critical number of infections is reached, natural herd immunity is achieved and the number of daily new infections starts to fall. But the daily total falls gradually, and the total number of infections in the epidemic depends on the height from which the daily total has to fall. Higher peaks take longer to descend from, leading to higher total numbers of infections.

6 I don’t think precise thresholds for “significant” or “immediate” are possible. These facts may be sources of debate. I do think COVID-19 unambiguously presented the UK with a threat of this type.

7 See Pitkin (1967) for an influential discussion of norms of accountability. For more recent discussions, see Grant and Keohane (2005), Mansbridge (2009). For an argument that accountability is not an essential feature of democracy, only a desirable one, see Landemore (2020).

8 This informal idea can be formalized in different ways by various decision-theoretic rules. See Voorhoeve (forthcoming) for an introduction to the decision-theoretic treatment of uncertainty.

9 Avian influenza viruses often evolve in a way that *increases* their pathogenicity. As Akhtar (2012, p. 106) notes, “All avian influenzas start off as mild, low-pathogenic viruses. However, once they enter domestic bird populations, they can rapidly mutate into highly pathogenic viruses.” Once a respiratory virus enters a large population of close-packed individuals, it is under selection for reproducing fast and being emitted fast by its host—not selection for sparing its host’s life. For discussion of the relevant selection pressures, see Sober (2020).
